# Supplementary figures and images for: Immunohistochemistry guided segmentation of benign epithelial cells, in situ lesions, and invasive epithelial cells in breast cancer slides
Source: PLoS One. 2025 Jul 17;20(7):e0328033. doi: 10.1371/journal.pone.0328033 (PMC12270177; doi:10.1371/journal.pone.0328033)

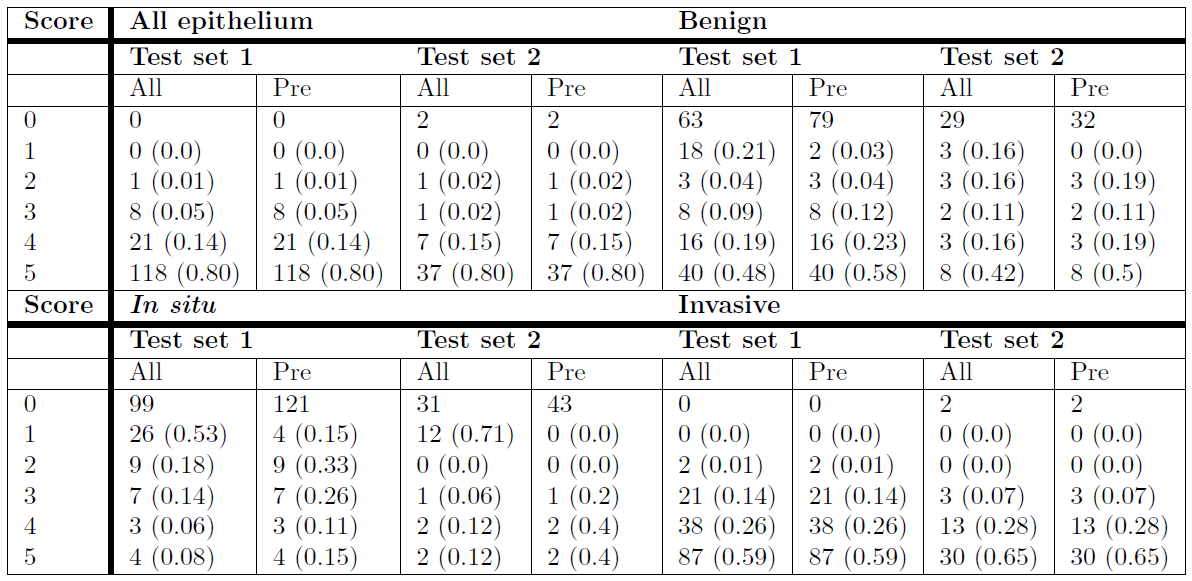

Supplement: S1 Table — Number of cases assigned to each score for each class (all epithelium, benign, in situ, and invasive). The scores under “Pre” represent scores where only cores with the respective class in the ground truth are included, otherwise the score is set to zero. The numbers in parentheses represent the percentage excluding score zero.) (TIFF) [file pone.0328033.s001.tiff]

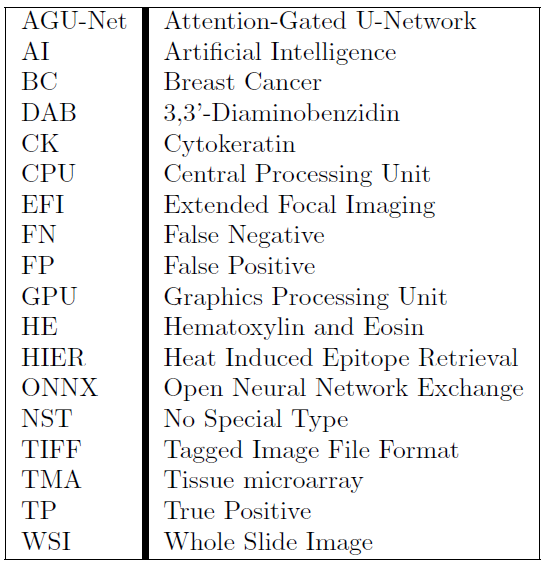

Supplement: S2 Table — (TIFF) [file pone.0328033.s002.tiff]
